# Supplementary material for: A degron-mimicking molecular glue drives CRBN homo-dimerization and degradation
Source: Nat Commun. 2025 Nov 19;16:10157. doi: 10.1038/s41467-025-65094-3 (PMC12630686; doi:10.1038/s41467-025-65094-3)

SUPPLEMENTARY INFORMATION

Title: A degron-mimicking molecular glue drives CRBN homo-dimerization and degradation

Author list: Gerasimos Langousis[1], Pablo Gainza[1], Moritz Hunkeler[2], Despoina Kapsitidou[1], Etienne J. Donckele[1], Stefano Annunziato[1], Lars Wiedmer[1], Katherine F. M. Jones[1], Bradley DeMarco[1], Chao Quan[1], Richard D. Bunker[1], Kevin J. Lumb[1], Bernhard Fasching[1], John C. Castle[1], Sharon A. Townson[1], Débora Bonenfant[1]*

Affiliations:

[1]Monte Rosa Therapeutics AG, Klybeckstrasse 191, WKL-136.3, 4057 Basel, Switzerland

[2]University of Basel, BioEM Lab, Mattenstrasse 26, 4058 Basel, Switzerland

*Corresponding author email: dbonenfant@monterosatx.com

Supplementary Figures

Supplementary Figure 1. Global quantitative proteomics in Jurkat cells. 3

Supplementary Figure 2. Biochemical thalidomide CRBN displacement assay. 4

Supplementary Figure 3. SEC-MALS analysis of CRBN-DDB1.. 5

Supplementary Figure 4. Data processing. 6

Supplementary Figure 5. Data and model quality. 7

Supplementary Figure 6. MGD and PPI footprints.. 8

Supplementary Figure 7. Proteomics in mouse cells. 9

Supplementary Figure 8. NanoBRET assay for CRBN mutants upon MRT-31619 treatment. ...10

Supplementary Figure 9. Western blot analysis of CRBN expression in Jurkat cells. 11

Supplementary Figure 10. DDB1 is not ubiquitinated upon MRT-31619 treatment. 12

Supplementary Figure 11. Model of the CRBN:CRBN structure with MRT-31619 superimposed to the CUL4/DDB1/E2 complex (PDB id: 8B3G)..13

Supplementary Table 1. Cryo-EM data collection, refinement and validation statistics. 14

Supplementary Methods……………………………………………………………………………..16

Western blots for Supplementary Figures…………………………………………………………..22

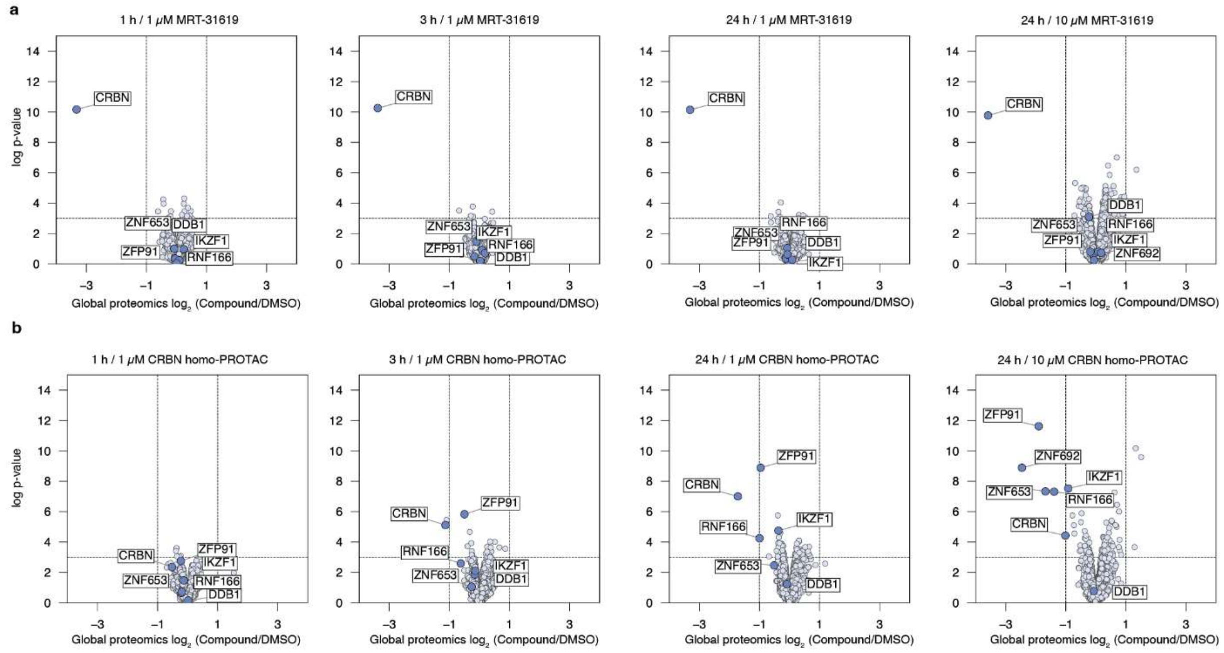

Supplementary Figure 1. Global quantitative proteomics in Jurkat cells.

Volcano plots show compound/DMSO protein abundance upon treatment with 1 μM of MRT-31619 **(a)** or CRBN homo-PROTAC **(b)** for 1 h, 3 h and 24 h, and with 10 μM of MRT-31619 **(a)** or CRBN homo-PROTAC for 24 h **(b)**. Proteins with at least 2 peptide features are displayed on the volcano plots. The protein ZNF692, detected with only 1 feature under the 1 μM conditions, is not displayed in the corresponding plots.

a

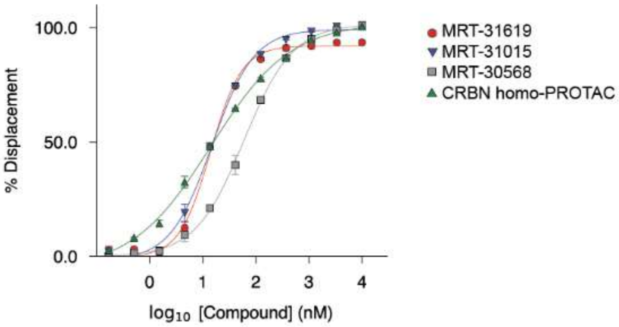

b

Compound	K_i (nM)	IC_{50} (nM)
MRT-30568	33	60.2
MRT-31015	8.23	15
MRT-31619	7.72	14.1
CRBN homo-PROTAC	7.93	14.3

Supplementary Figure 2. Biochemical thalidomide displacement assay.

Activity for displacing thalidomide from CRBN was monitored in a TR-FRET assay. (**a**) Curves and (**b**) CRBN IC_{50} and K_i values for MRT-31619, MRT-30568, MRT-31015 and CRBN homo-PROTAC. Two biological replicates were plotted as mean ± SD.

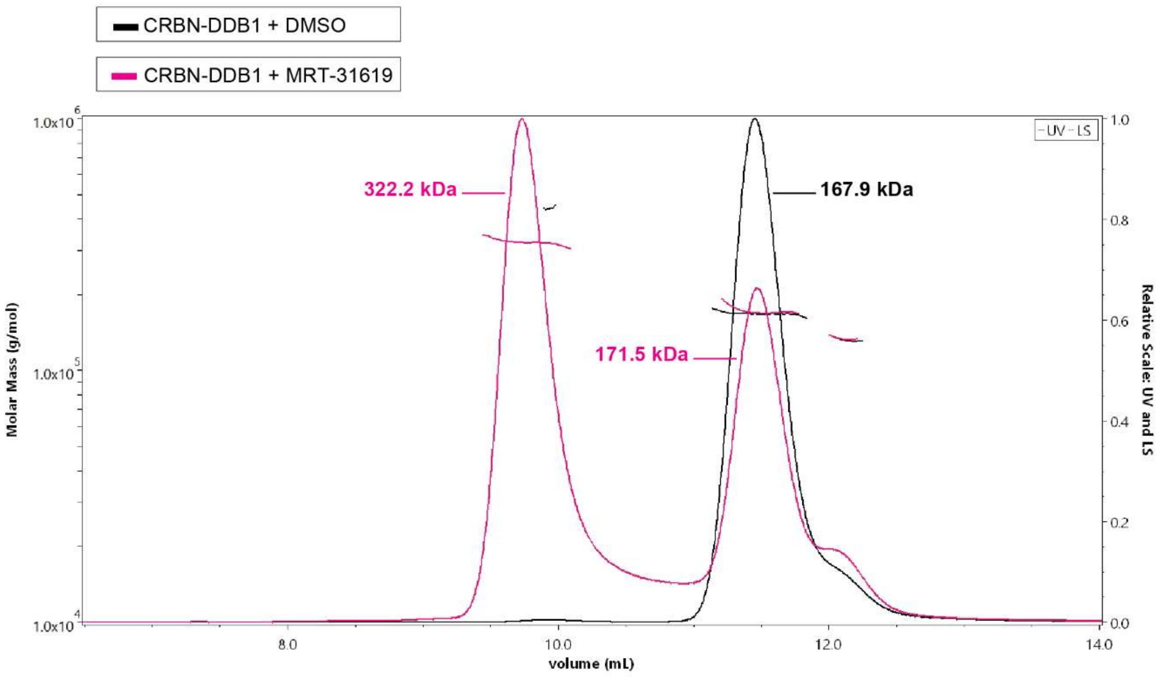

Supplementary Figure 3. SEC-MALS analysis of CRBN-DDB1.

Purified recombinant CRBN-DDB1 were subjected to size exclusion chromatography (SEC) coupled to multiangle light scattering (MALS) analysis upon treatment with MRT-31619 (and DMSO as control). Calculated molecular weights of prominent peaks are indicated. Expected molecular weights are: CRBN (46.1 kDa), DDB1 (127 kDa), CRBN-DDB1 (173.1 kDa), CRBN-DDB1-CRBN-DDB1 (346.2 kDa).

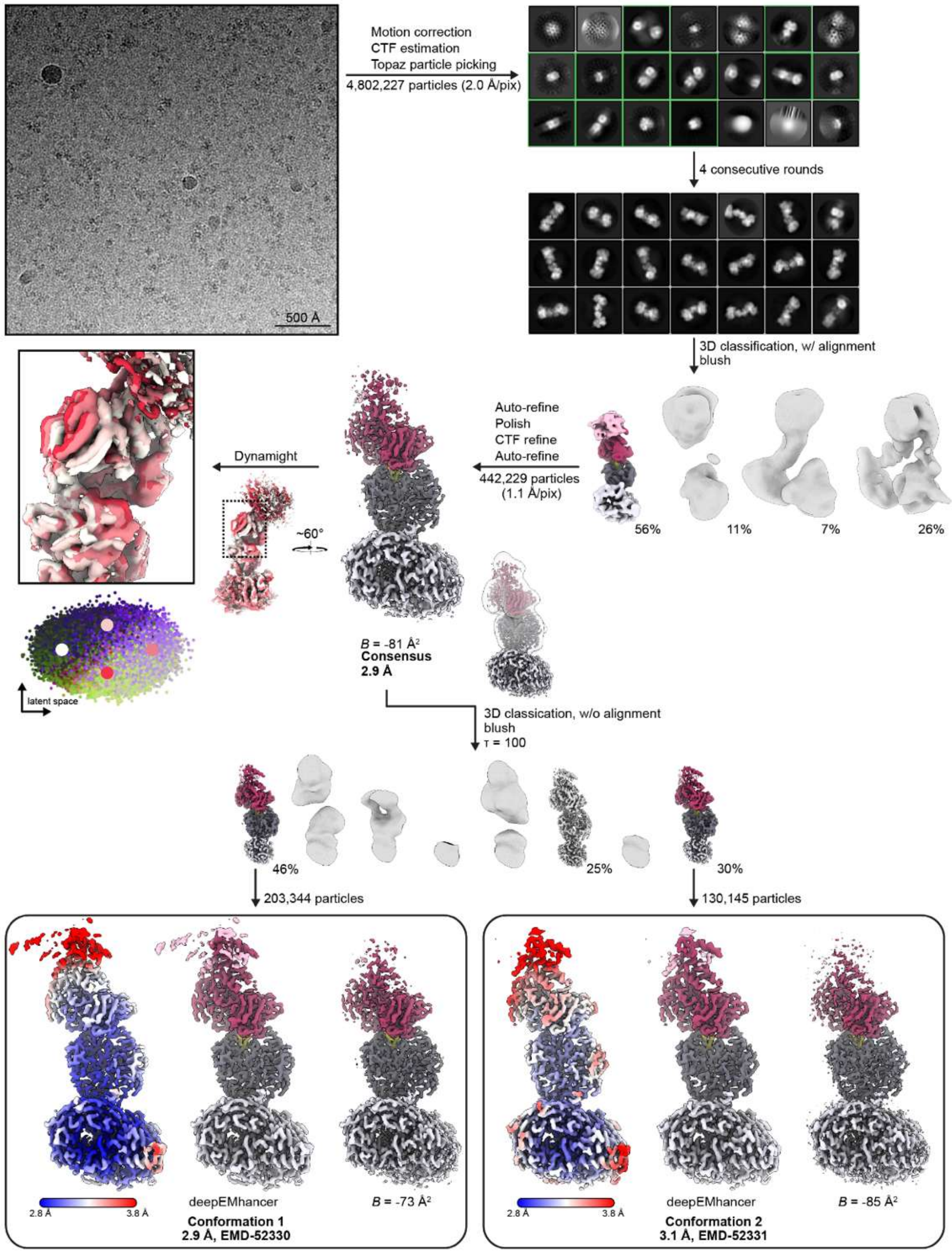

Supplementary Figure 4. Data processing.

Overview of data processing from raw micrograph (low pass filtered to 10 Å, scale bar indicated) to final maps. Maps post-processed and sharpened in Relion and deepEMhancer are shown, respectively, as well as maps colored according to local resolution (resolution mapped onto maps from deepEMhancer). For all classifications, particles belonging to colored densities were taken into subsequent steps of processing. Dynamight analysis on the particles from the consensus refinement revealed substantial residual conformational heterogeneity. Four volumes representing four different coordinates in latent space are shown as overlay, colored from white to red.

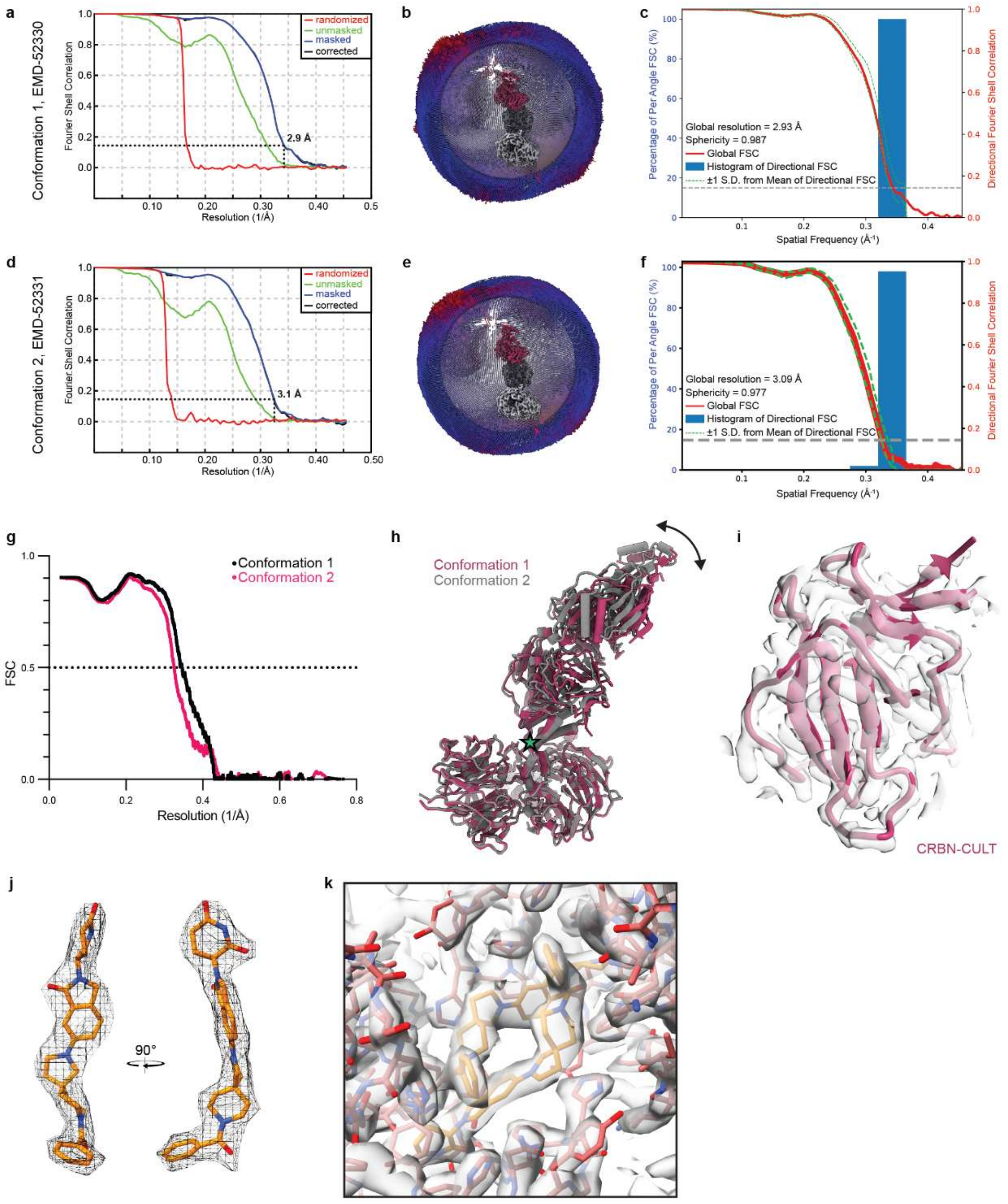

Supplementary Figure 5. Data and model quality.

a,d, The FSC curves, **b,e,** viewing distribution, and **c,f,** 3DFSC plots of the 2 identified conformations **g,** Model-to-map FSC curves for the two conformations. **h,** Superposition of the conformations, illustrating the kink motion in the CRBN attachment (green star). The motion is indicated with an arrow. **i,** Density example for the CRBN-CULT domain. j) Density of the MRT-31619 (in map from conformation 1). **k,** Density of MRT-31619 with density of surrounding residues at same threshold level as in j) and approximately same orientation as in Figure 2.

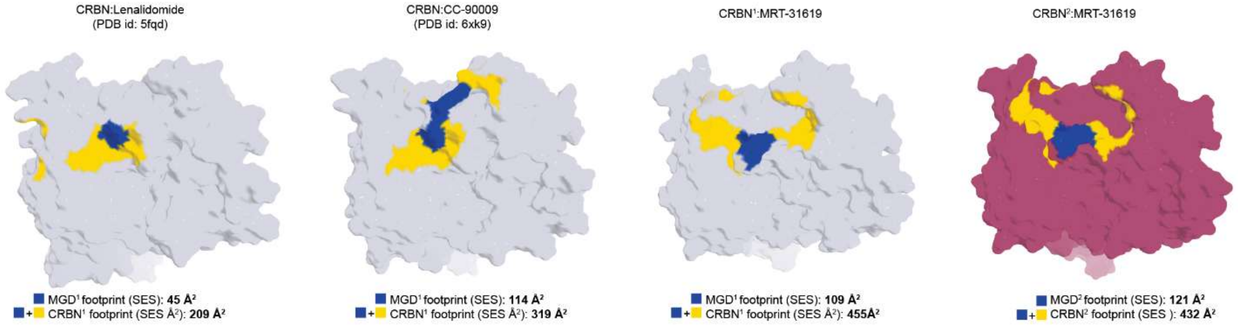

Supplementary Figure 6. MGD and PPI footprints.

Footprints for IKZF1: Lenalidomide, GSPT1:CC90009, CRBN: MRT-31619: protomer 1 ($CRBN^1$) and protomer 2 ($CRBN^2$). MGD footprints in blue and PPI footprints in yellow.

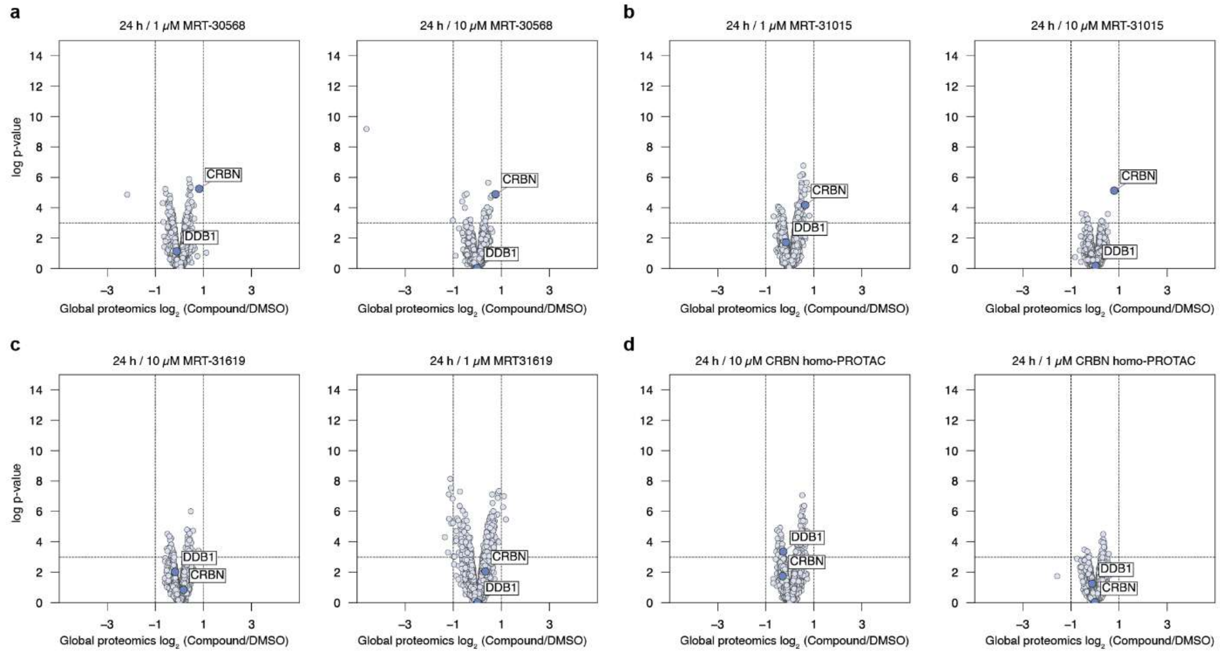

Supplementary Figure 7. Proteomics in mouse cells.

Volcano plots show compound/DMSO protein abundance upon treatment for 24 h with 1 μM or 10 μM of MRT-30568 (**a**) MRT-31015 (**b**) MRT-31619 (**c**) and CRBN homo-PROTAC (**d**). Proteins with at least 2 peptide features are displayed on the volcano plots.

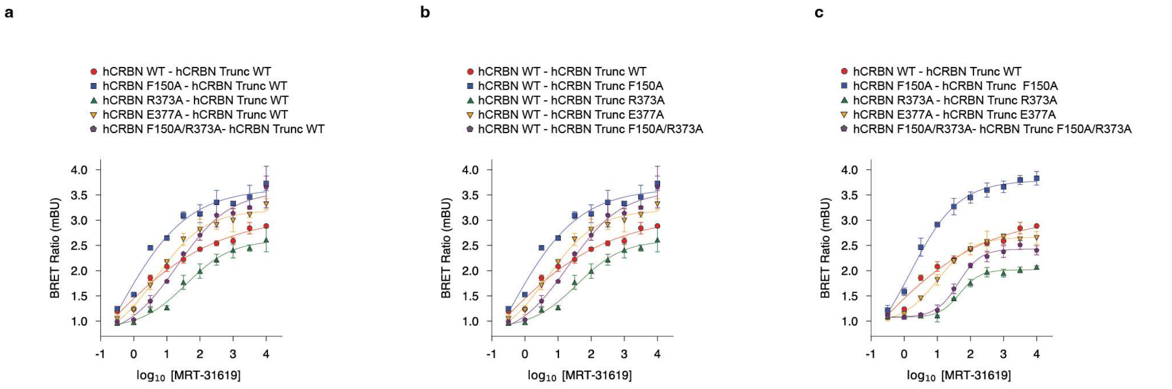

Construct	EC_{50} (nM)
hCRBN WT - hCRBN Trunc WT	6.83
hCRBN F150A - hCRBN Trunc WT	4.83
hCRBN R373A - hCRBN Trunc WT	6.15
hCRBN E377A - hCRBN Trunc WT	2.95
hCRBN F150A/R373A- hCRBN Trunc WT	4.37

Construct	EC_{50} (nM)
hCRBN WT - hCRBN Trunc WT	6.83
hCRBN WT - hCRBN Trunc F150A	3.27
hCRBN WT - hCRBN Trunc R373A	42.63
hCRBN WT - hCRBN Trunc E377A	7.82
hCRBN WT - hCRBN Trunc F150A/R373A	27

Construct	EC_{50} (nM)
hCRBN WT - hCRBN Trunc WT	6.83
hCRBN F150A - hCRBN Trunc F150A	3.36
hCRBN R373A - hCRBN Trunc R373A	45.77
hCRBN E377A - hCRBN Trunc E377A	10.48
hCRBN F150A/R373A- hCRBN Trunc F150A/R373A	41.9

Supplementary Figure 8. NanoBRET assay for CRBN mutants upon MRT-31619 treatment.

a, Wild type (WT) and mutant HaloTag-hCRBN constructs are indicated. **b**, WT and mutant hCRBN-NanoLuc constructs are indicated. **c**, WT and mutant HaloTag-hCRBN and hCRBN-NanoLuc constructs are indicated. For these experiments, hCRBN-NanoLuc constructs with the 191-248 aa deletion (Trunc) that do not bind DDB1 were used. Three biological replicates were plotted as mean ± SEM.

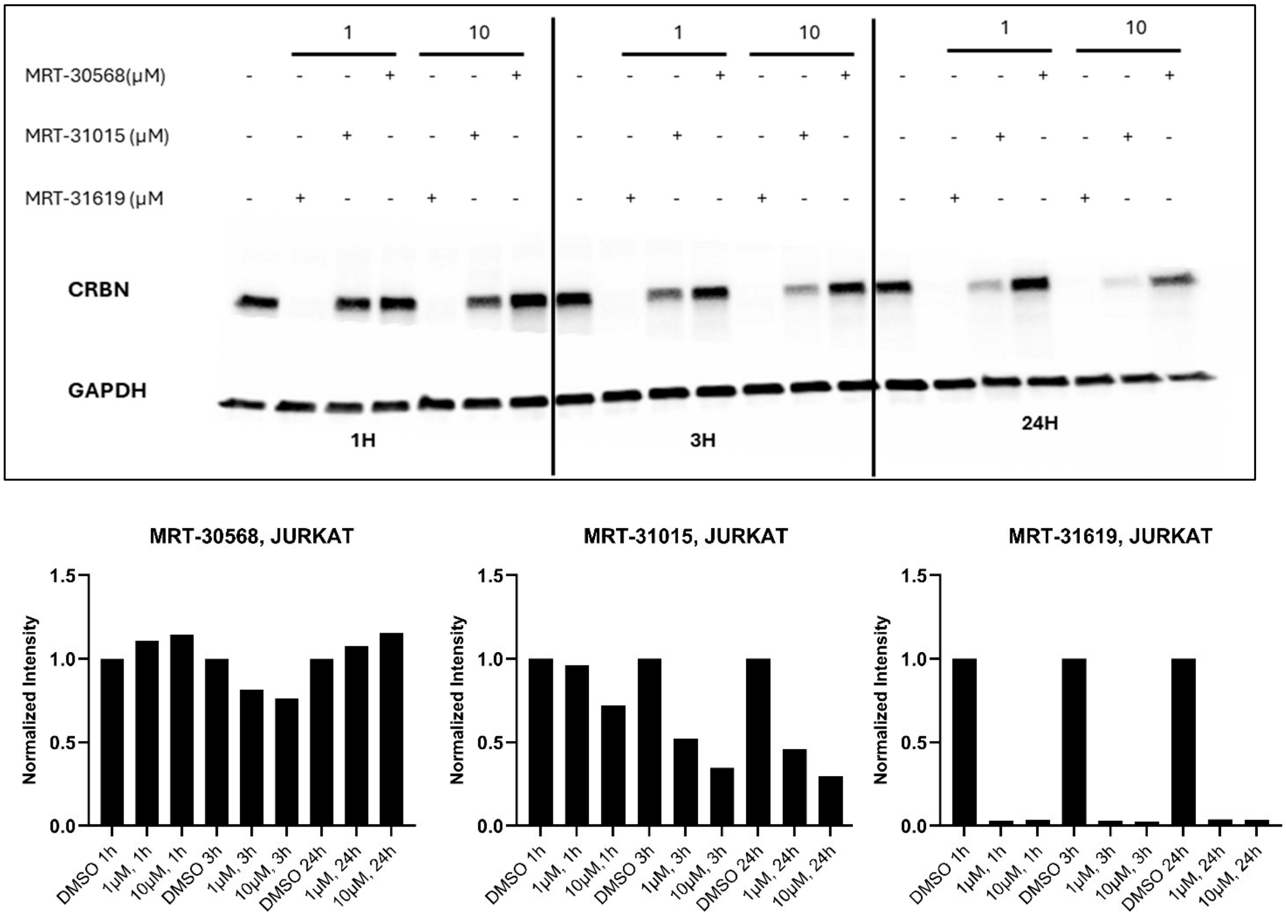

Supplementary Figure 9. Western blot analysis of CRBN abundance in Jurkat cells.

Cells were treated with either 1 µM or 10 µM of MRT-31619, MRT-31015 or MRT-30568 for 1 h, 3 h or 24 h. GAPDH was used as loading control. Bar graph representations of CRBN band intensities normalized to GAPDH. Results are representative of 2 independent experiments.

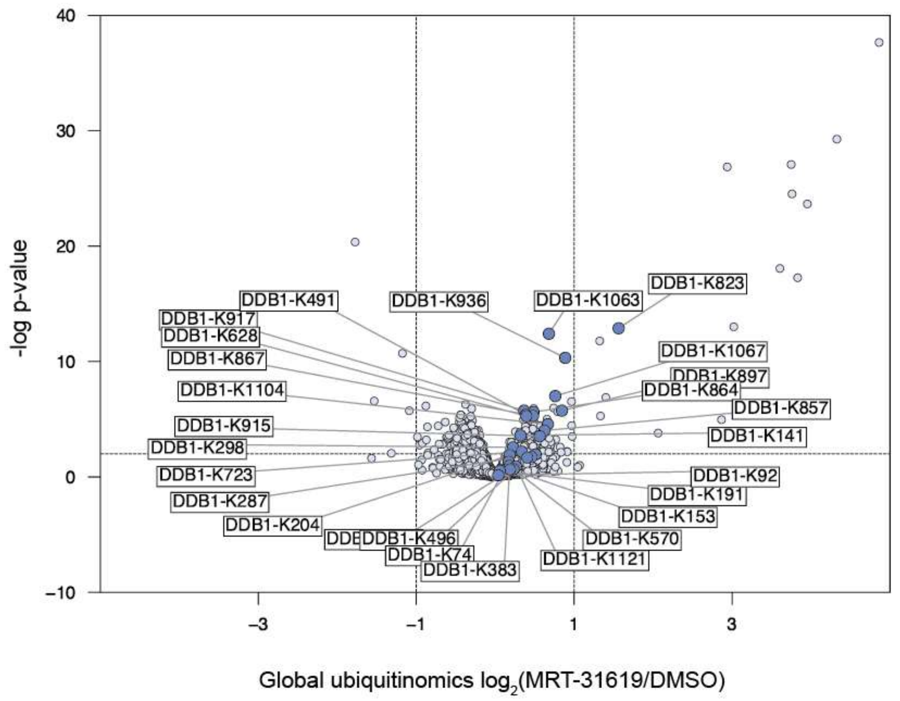

Supplementary Figure 10. Ubiquitinomics data labeling the DDB1 ubiquitinated peptides.

Global ubiquitinomics in Jurkat cells. Volcano plots show compound/DMSO K-GG peptide abundance upon treatment with 10 μM of MRT-31619 for 30 min.

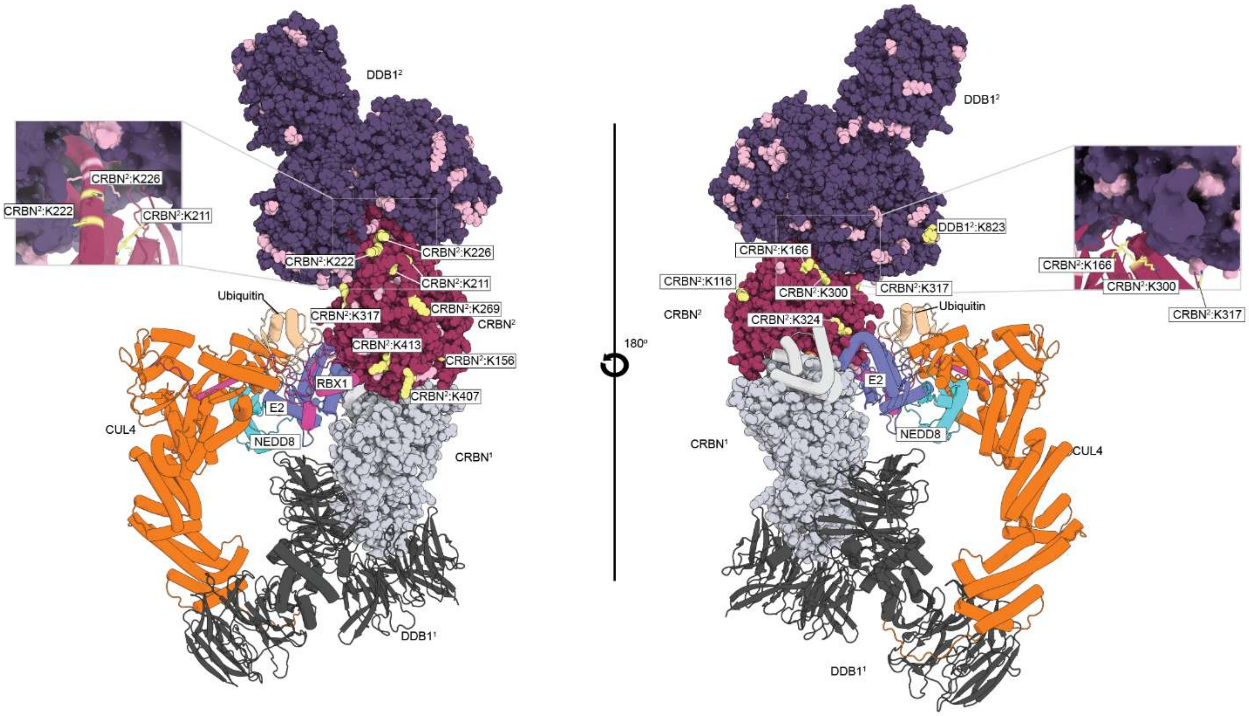

Supplementary Figure 11. Model of the CRBN:CRBN structure with MRT-31619 superimposed to the CUL4/DDB1/E2 complex (PDB id: 8B3G).

Ubiquitinylated lysines in CRBN^2 and DDB1^2 are shown in yellow. Lysines in CRBN^2 and DDB1^2 that are not ubiquitinylated are shown in pink. The most significantly affected ubiquitinated lysine residues in CRBN^2 and DDB1^2 are labeled.

Supplementary Table 1. Cryo-EM data collection, refinement and validation statistics.

	Data collection	
**Microscope**	Thermo Fisher Scientific Titan Krios G4	
**Voltage (kV)**	300	
**Camera**	Falcon4i	
**Energy filter (slit width, eV)**	Selectris X (10)	
**Acquisition software**	EPU v3.8.1	
**Magnification**	165,000x	
**Defocus range (μm)**	-0.8 - -2	
**Pixel size (Å/pix)**	0.73	
**Electron exposure (e^{-}/Å^2)**	40	
**Micrographs**	20,582	
**Initial no. of particles**	4,802,227	
	Conformation 1	Conformation 2
**Final no. of particles**	203,344	130,145
**Map symmetry**	C1	C1
**Map resolution (Å) (FSC threshold 0.143)**	2.93	3.09
**Resolution range (Å)**	2.5-3.7	2.7-4.2
**3DFSC Sphericity**	0.987	0.977
	**Model composition**	
**Protein (residues)**	1506	1499
**Ligand**<br>Zn<br>MRT0031619	 2<br>2	 2<br>2
	**Model Refinement**	
**Refinement package**	Phenix.real_space_refine	Phenix.real_space_refine
**Resolution cutoff**	2.9	2.9
**Model-Map CC**	0.70	0.64
**Model-Map FSC (0.5)**	2.9	3.1
***B* factors (Å^2)**<br>Protein residues<br>Ligands	 98<br>51	 108<br>51
**R.m.s deviations from ideal values**<br>Bond lengths (Å)<br>Bond angles (°)	 0.003<br>0.527	 0.003<br>0.557
	**Model Validation**	
**Molprobity score**	1.50	1.59
**CaBLAM outliers (%)**	1.45	1.05
**Clash score**	3.24	4.17
**Rotamer outliers (%)**	2.09	1.50
**C_β outliers (%)**	N/A	N/A
**Ramachandran plot (%)**<br>Favored<br>Allowed<br>Disallowed	 97.22<br>2.78<br>0	 96.18<br>3.82<br>0
**EMRinger score**	3.45	3.54

Supplementary Methods

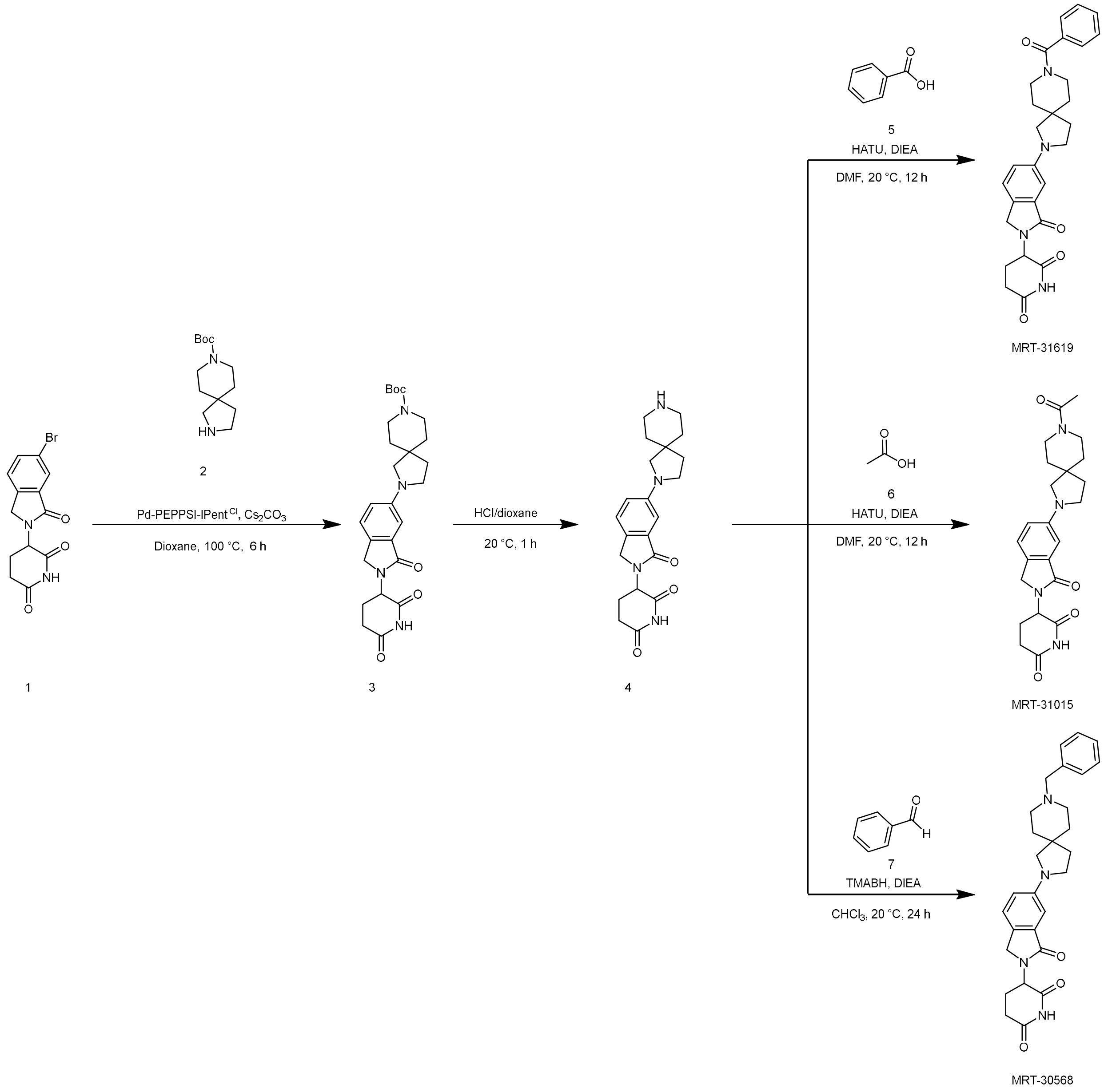

Synthesis of MRT-31619, MRT-31015 and MRT-30568

A mixture of 3-(6-bromo-1-oxo-isoindolin-2-yl)piperidine-2,6-dione (**1** ; 0.800 g, 2.48 mmol, 1.00 eq), tert-butyl 2,8-diazaspiro[4.5]decane-8-carboxylate (**2**; 892 mg, 3.71 mmol, 1.50 eq), 1,3-bis[2,6-bis(1-ethylpropyl)phenyl]-4,5-dichloro-2H-imidazol-1-ium-2-ide; 3-chloropyridine;dichloropalladium (213 mg, 247 µmol, 0.100 eq), cesium carbonate (1.61 g, 4.95 mmol, 2.00 eq) in dioxane (10.0 mL) was degassed and purged with nitrogen for 3 times, and then the mixture was stirred at 100 °C for 6 h under nitrogen atmosphere. The reaction mixture was filtered, and the filtrate was concentrated under reduced pressure to give a residue. The residue was purified by Prep-HPLC (column: Phenomenex luna C18 150*25 mm* 10 µm; mobile

phase: [water (0.225% formic acid) - acetonitrile]; gradient: 22%-52% B over 9.0 min to give desired tert-butyl 2-(2-(2,6-dioxopiperidin-3-yl)-3-oxoisoindolin-5-yl)-2,8-diazaspiro[4.5]decane-8-carboxylate (**3**; 400 mg, 580 µmol, 23% yield, 70% purity) as a white solid.

To a solution of compound **3** (400 mg, 828 µmol, 1.00 eq) in hydrochloride/1,4-dioxane (2.00 M, 50.0 mL, 120 eq) was stirred at 20 °C for 1 h. The reaction mixture was concentrated under reduced pressure to give Compound 3-(1-oxo-6-(2,8-diazaspiro[4.5]decan-2-yl)isoindolin-2-yl)piperidine-2,6-dione (**4**; 300 mg, 627 µmol, 75% yield, 80% purity) as a white solid.

Step 3. MRT-31619. To a solution of **4** (150 mg, 392 µmol, 1.00 eq) 4 and benzoic acid (**5**, 71.8 mg, 588 µmol, 89.8 µL, 1.50 eq) in *N,N*-dimethylformamide (2.00 mL) was added 2-(3H-[1,2,3]triazolo[4,5-b]pyridin-3-yl)-1,1,3,3-tetramethyluronium hexafluorophosphate (223 mg, 588 µmol, 1.50 eq) and *N*-ethyl-*N*-isopropylpropan-2-amine (152 mg, 1.18 mmol, 204 µL, 3.00 eq). The mixture was stirred at 20 °C for 12 h. The reaction mixture was filtered to give the filtrate. The residue was purified by Prep-HPLC (column: Phenomenex luna C18 150*25mm* 10µm;mobile phase: [water(0.225% formic acid)- acetonitrile]; gradient:28%-58% B over 9.0 min) to give Compound 3-(6-(8-benzoyl-2,8-diazaspiro[4.5]decan-2-yl)-1-oxoisoindolin-2-yl)piperidine-2,6-dione (**MRT-31619**; 98.62 mg, 200 µmol, 25% yield, 99% purity) as an off-white solid. ^{1}H NMR (400 MHz, DMSO-d_6) δ = 10.96 (s, 1H), 7.48 - 7.43 (m, 3H), 7.42 - 7.36 (m, 3H), 6.88 - 6.73 (m, 2H), 5.09 (dd, J = 5.0, 13.3 Hz, 1H), 4.37 - 4.27 (m, 1H), 4.25 - 4.12 (m, 1H), 3.84 - 3.69 (m, 1H), 3.65 - 3.53 (m, 1H), 3.43 - 3.34 (m, 4H), 3.25 (br s, 2H), 2.97 - 2.86 (m, 1H), 2.61 - 2.56 (m, 1H), 2.41 - 2.31 (m, 1H), 2.04 - 1.90 (m, 3H), 1.70 - 1.45 (m, 4H). ^{13}C NMR (101 MHz, DMSO-d6) δ = 173.42, 171.61,169.37, 169.27, 148.27, 136.85, 132.99, 129.82, 128.90, 127.12, 124.20, 116.18, 104.78, 57.98, 52.07, 46.99, 46.62, 45.27, 41.10, 35.86, 35.46, 34.82 31.70, 22.99. MS (ESI) m/z 487.4 $[M+H]^+$.

Step 3. MRT-31015. To a solution of **4** (150 mg, 392 µmol, 1.00 eq) and acetic acid (**6**, 35.2 mg, 588 µmol, 34 µL, 1.50 eq) in *N,N*-dimethylformamide (2.00 mL) was added 2-(3H-[1,2,3]triazolo[4,5-b]pyridin-3-yl)-1,1,3,3-tetramethyluronium hexafluorophosphate (223 mg, 588 µmol, 1.50 eq) and *N*-ethyl-*N*-isopropylpropan-2-amine (152 mg, 1.18 mmol, 204 µL, 3.00 eq). The mixture was stirred at 20 °C for 12 h. The reaction mixture was filtered to give the filtrate. The residue was purified by Prep-HPLC (column: Phenomenex luna C18 150*25mm* 10µm;mobile phase: [water(0.225% formic acid)- acetonitrile]; gradient:28%-58% B over 9.0 min) to give Compound 3-(6-(8-benzoyl-2,8-diazaspiro[4.5]decan-2-yl)-1-oxoisoindolin-2-yl)piperidine-2,6-dione (**MRT-31615**; 44.9 mg, 106 µmol, 27% yield, 97% purity) as an off-white solid. ^{1}H NMR (400 MHz, DMSO-d6) δ = 11.26 - 10.66 (m, 1H), 7.37 (d, J = 8.4 Hz, 1H), 6.89 - 6.73 (m, 2H), 5.09 (dd, J = 5.0, 13.4 Hz, 1H), 4.34 - 4.26 (m, 1H), 4.22 - 4.12 (m, 1H), 3.59 - 3.39 (m, 4H), 3.36 (br s, 1H), 3.30 (br s, 1H), 3.21 (s, 2H), 2.98 - 2.85 (m, 1H), 2.61 - 2.54 (m, 1H), 2.37 (dt, J = 9.1, 13.1Hz, 1H), 2.05 - 1.95 (m, 4H), 1.90 (br t, J = 6.9 Hz, 2H), 1.63 - 1.39 (m, 4H). 1H NMR (400 MHz, DMSO-d6+D2O) δ = 7.36 (d, J = 8.3 Hz, 1H), 6.86 - 6.79 (m, 1H), 6.76 (d, J = 1.8 Hz, 1H), 4.98 (dd, J = 5.0, 13.3 Hz, 1H), 4.39 - 4.26 (m, 1H), 4.20 - 4.11 (m, 1H), 3.53 - 3.38 (m, 4H), 3.31 (br t, J = 6.6 Hz, 2H), 3.15 (s, 2H), 2.88 - 2.77 (m, 1H), 2.61 (br d, J = 15.9 Hz, 1H), 2.43 - 2.28 (m, 1H), 1.98 (s, 4H), 1.88 (br t, J = 6.8 Hz, 2H), 1.60 - 1.37 (m, 4H) . 1.88 (br t, J = 6.8 Hz, 2H), 1.60 - 1.37 (m, 4H). ^{13}C NMR (101 MHz, DMSO-d6) δ = 173.43, 171.63, 169.27, 168.37, 158.91, 148.27, 132.99, 128.86, 124.19, 116.17, 104.75, 58.01, 52.05, 46.97, 46.61, 43.88, 40.92, 38.93, 35.81, 35.57, 34.83, 31.70, 23.00, 21.83. MS (ESI) m/z 425.2 $[M+H]^+$

Step 3. MRT-30568. To a solution of **4** (150 mg, 392 µmol, 1.00 eq), benzaldehyde (**7**, 50 mg, 470.4 µmol, 48 µL 1.2 eq) in *N*,*N*-Diisopropylethylamine (2.00 mL) and chloroform (1.05 mL) was

added bis(acetyloxy)boranuidyl acetate; tetramethylazanium (412 mg, 4.0 eq). The mixture was stirred at 20 °C for 24 h. The solvent was evaporated under reduced pressure and filtered. The residue was purified by Prep-HPLC (column: Chromatorex 18 SMB 100-5T (Waters) using gradient from 15% to 25% of phase B in phase A (A - 0.1% trifluoroacetic acid in water, B - 0.1% trifluoroacetic acid in the mixture of MeCN and water (vol% 95:5))) to give Compound 3-(6-(8-benzyl-2,8-diazaspiro[4.5]decan-2-yl)-1-oxoisoindolin-2-yl)piperidine-2,6-dione (**MRT-30568**; 50 mg, 106 μmol, 27% yield, 99% purity) as an off-white solid. ^{1}H NMR (400 MHz, DMSO-d6) δ = 10.97 (br d, J = 3.3 Hz, 1H), 7.49 (s, 5H), 7.43 - 7.35 (m, 1H), 6.88 - 6.74 (m, 2H), 5.18 - 4.97 (m, 1H), 4.41 - 4.26 (m, 3H), 4.24 - 4.13 (m, 1H), 3.40 (br d, J = 5.1 Hz, 2H), 3.31 - 3.27 (m, 2H), 3.20 - 3.08 (m, 3H), 2.97 - 2.84 (m, 1H), 2.59 (br dd, J = 3.2, 14.3 Hz, 2H), 2.43 - 2.32 (m, 1H), 2.07 - 1.94 (m, 2H), 1.91 - 1.71 (m, 5H). ^{1}H NMR (400 MHz, DMSO-d6+D2O) δ = 7.47 (s, 5H), 7.43 - 7.33 (m, 1H), 6.89 - 6.69 (m, 2H), 5.10 - 4.96 (m, 1H), 4.35 - 4.26 (m, 3H), 4.23 - 4.12 (m, 1H), 3.36 - 3.26 (m, 4H), 3.17 - 3.07 (m, 3H), 2.92 - 2.81 (m, 1H), 2.62 - 2.56 (m, 2H), 2.39 - 2.31 (m, 1H), 2.03 - 1.94 (m, 2H), 1.79 (br s, 5H). ^{13}C NMR (101 MHz, DMSO-d6) δ = 173.39, 171.59, 169.27, 163.95, 148.29, 137.35, 132.99, 129.72, 128.87, 128.76, 127.85, 124.19, 116.15, 104.70, 62.11, 52.09, 47.00, 39.95, 34.58, 31.70, 23.01. MS (ESI) m/z 473.3 $[M+H]^+$

QC data of **MRT-31619**. A: LCMS at 254 nm; B ^{1}H NMR (DMSO d_6); C: ^{13}C NMR (DMSO d_6)

A) LCMS

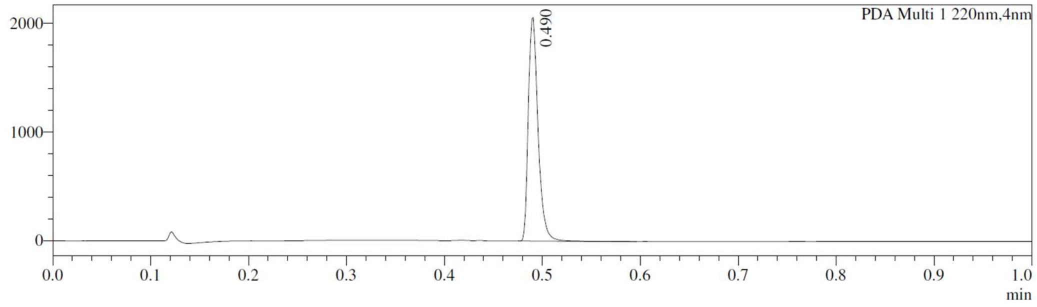

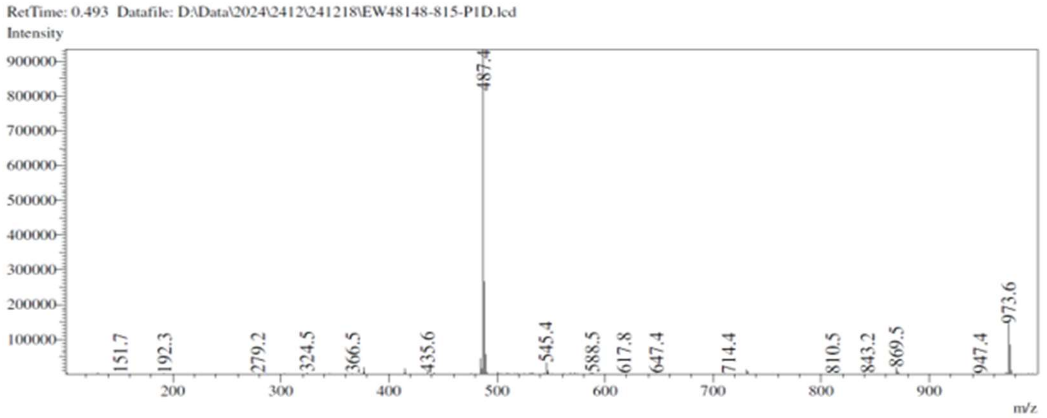

B) ^{1}H NMR

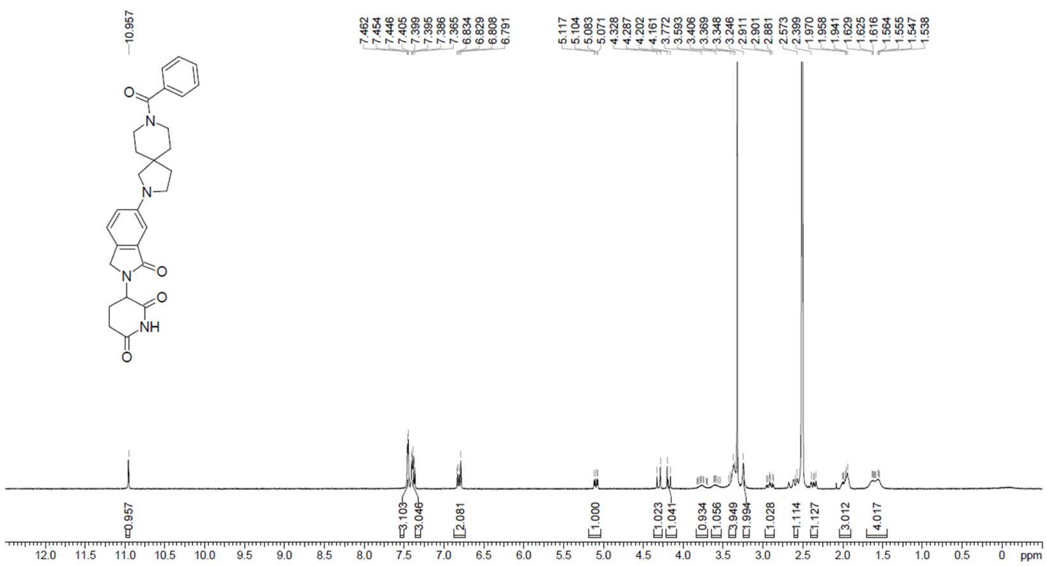

C) ^{13}C NMR

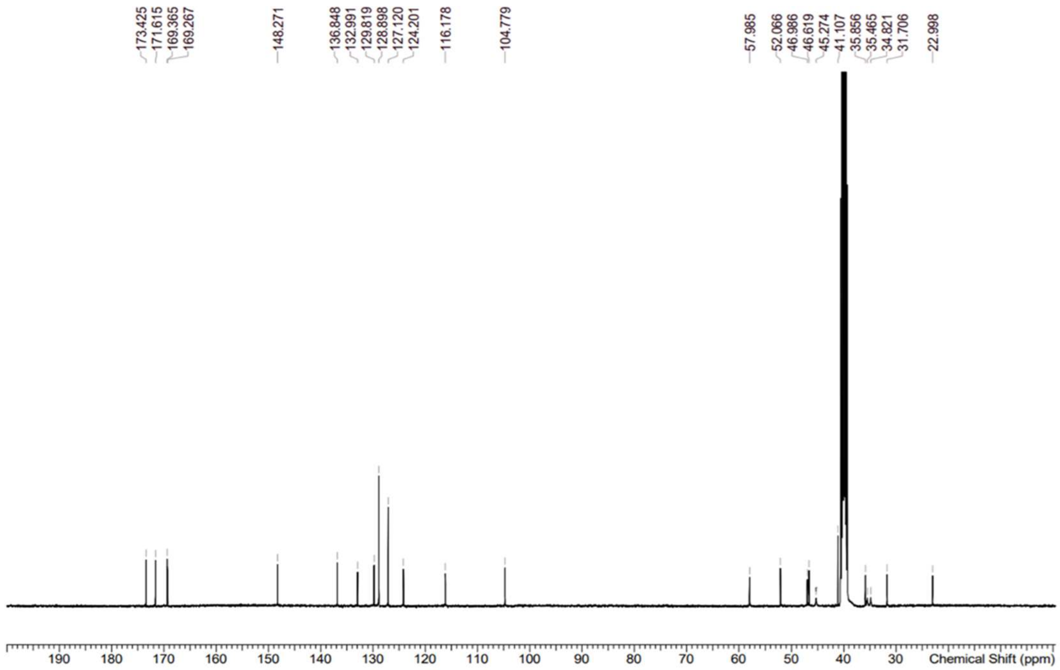

QC data of **MRT-31015**. A: LCMS at 254 nm; B ^{1}H NMR (DMSO d_6); C: ^{13}C NMR (DMSO d_6)

A. **LCMS**

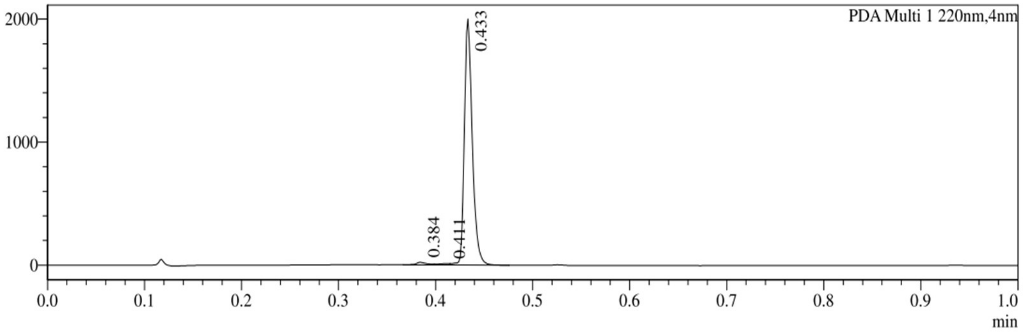

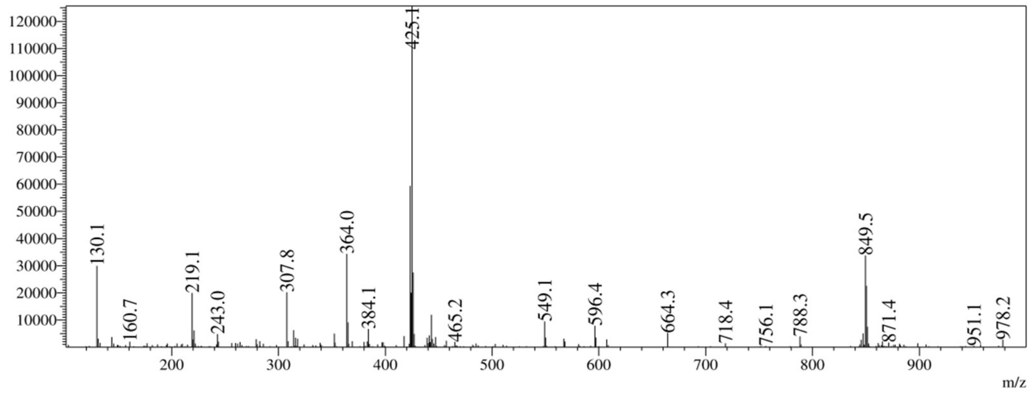

B) ^{1}H NMR

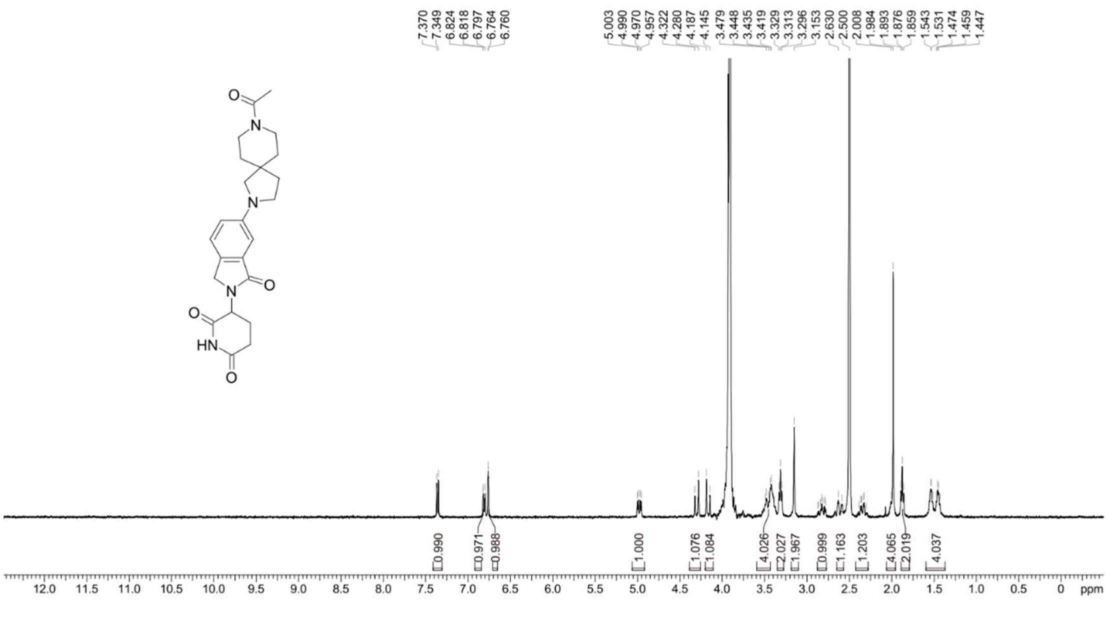

C) ^{13}C NMR

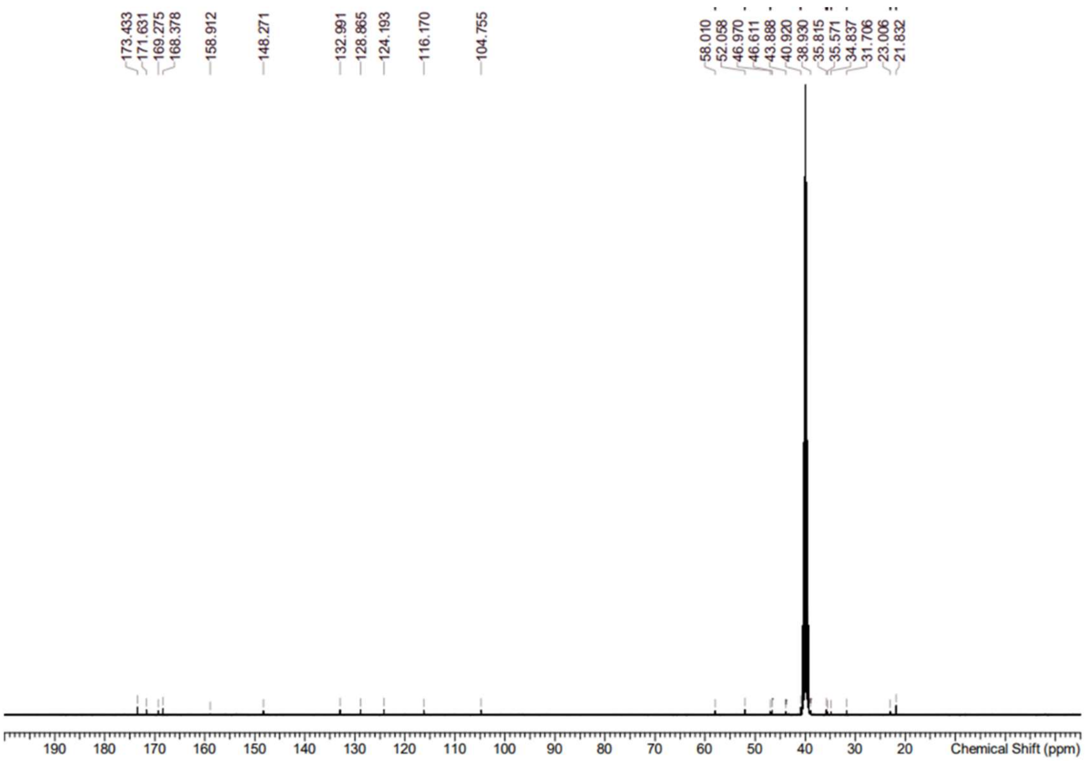

QC data of **MRT-30568**. A: LCMS at 254; B: 1H NMR (DMSO d[6])

A) LCMS

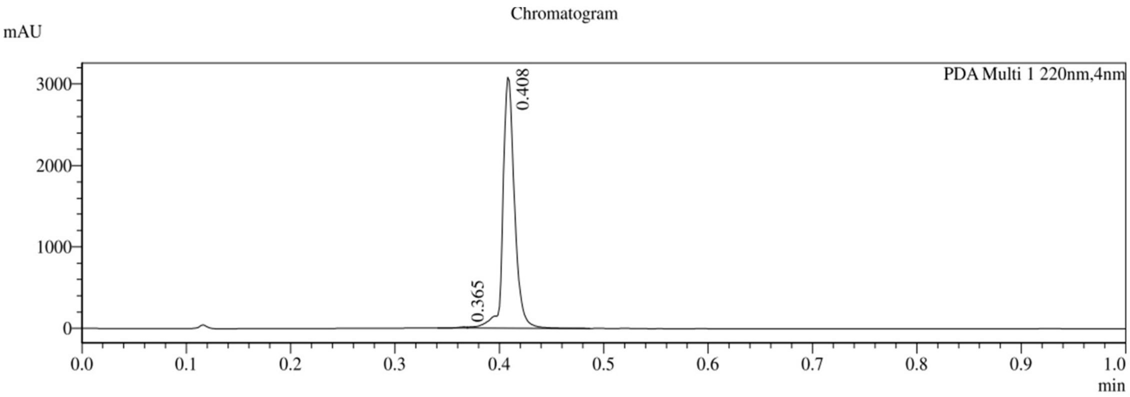

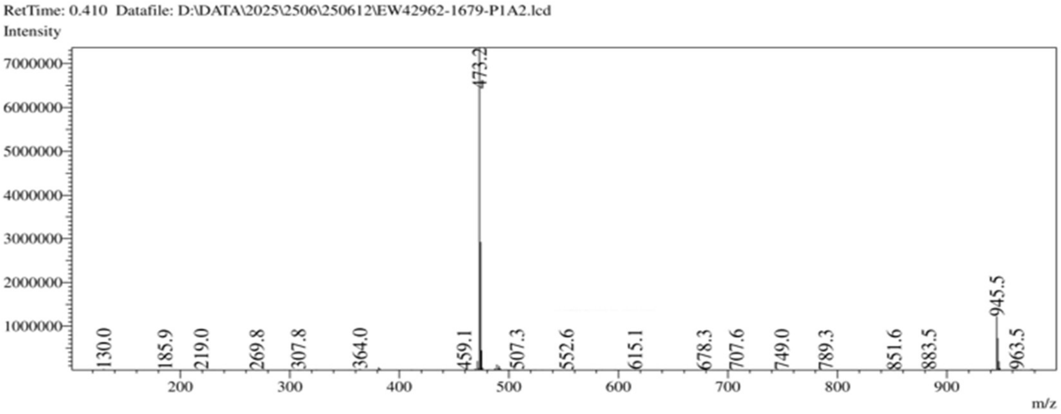
RetTime: 0.410 Datafile: D:\DATA\2025\2506\250612\EW42962-1679-P1A2.lcd
Intensity
473.2
945.5
963.5
m/z

B) ^{1}H NMR

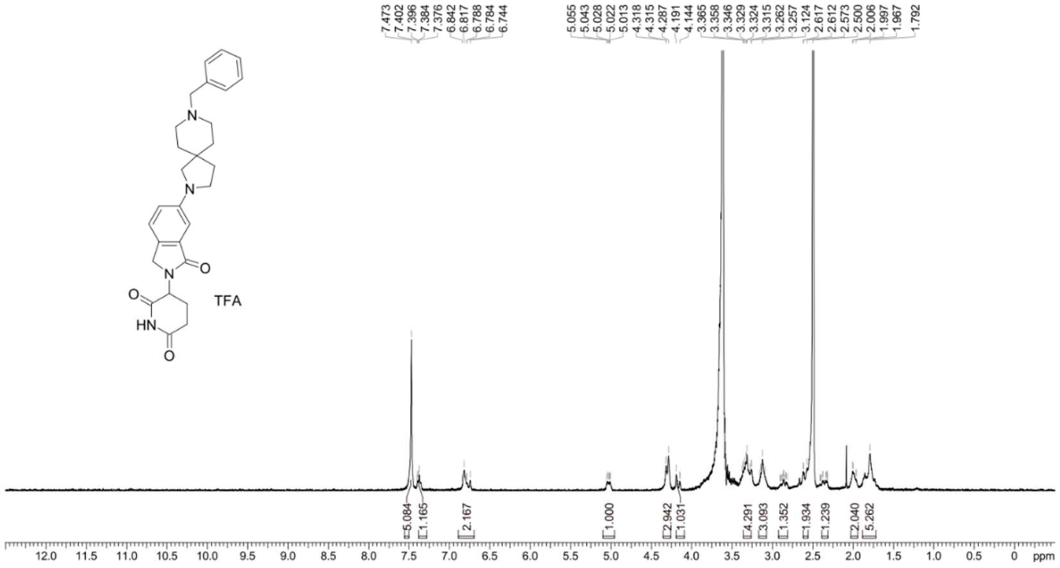
7.473
7.402
7.396
7.384
7.376
6.842
6.817
6.788
6.784
6.744
5.055
5.043
5.028
5.022
5.013
4.318
4.315
4.287
4.191
4.144
3.365
3.358
3.346
3.329
3.324
3.315
3.262
3.257
3.124
2.617
2.612
2.573
2.500
2.006
1.997
1.967
1.792
TFA
ppm

C) ^{13}C NMR

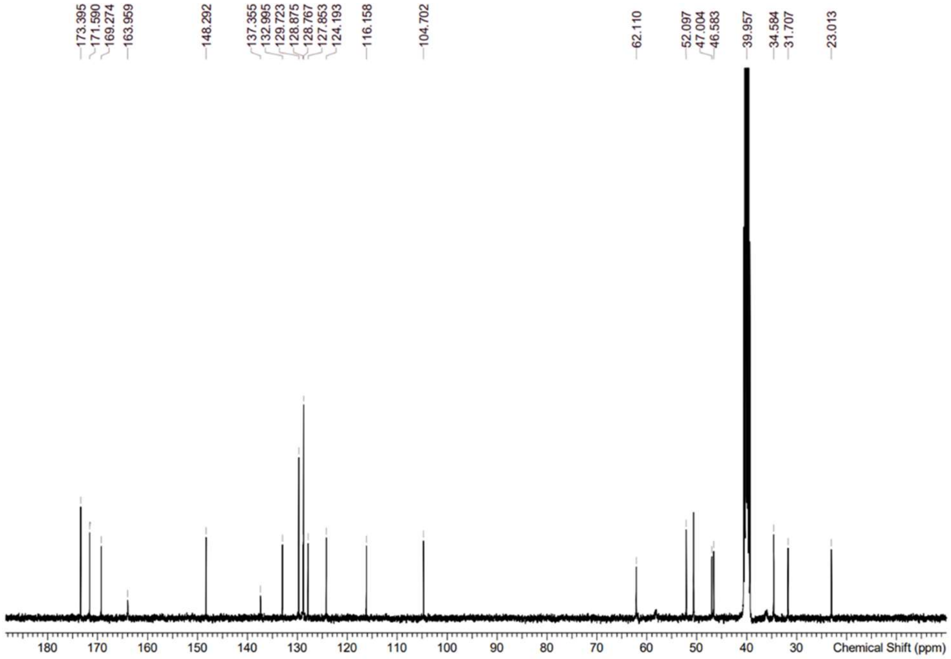

173.395
171.590
169.274
163.959
148.292
137.355
132.995
129.723
128.875
128.767
127.853
124.193
116.158
104.702
62.110
52.097
47.004
46.583
39.957
34.584
31.707
23.013
180
170
160
150
140
130
120
110
100
90
80
70
60
50
40
30
Chemical Shift (ppm)

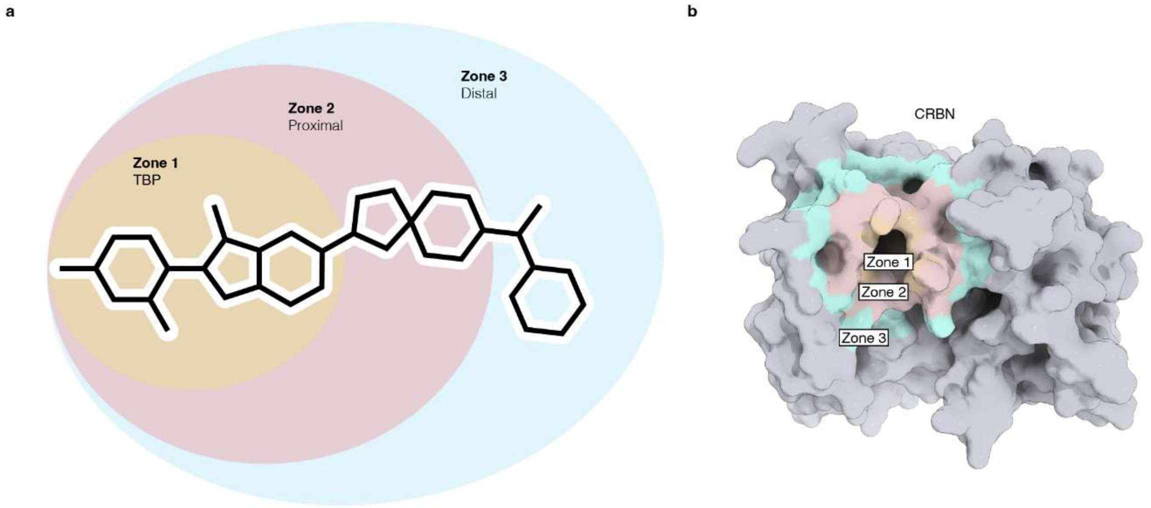

Size and design of the CRBN library

MRT-31619 and other compounds in this study came from a proprietary 50,000 compound library. The design of this library has been previously described[9]. **a**, briefly, Monte Rosa's design framework for CRBN-directed molecular glue degraders divides the compound's binding environment into three zones that guide rational optimization. Zone 1 is the thalidomide-binding pocket (TBP) within CRBN, where conserved interactions with the tri-Trp cage and surrounding residues are essential for degron recognition but not sufficient for neosubstrate selectivity. Zone 2, the proximal region just beyond the TBP, is the key determinant of selectivity, as even small chemical changes here can switch substrate preference by modulating interactions with degron sidechains. Zone 3, the distal region, further enhances CRBN affinity and fine-tunes specificity, especially for neosubstrates with extended protein–protein interfaces. **b**, together, these zones establish design rules: Zone 1 interactions must be conserved for CRBN binding, Zone 2 modifications dictate neosubstrate selectivity, and Zone 3 modifications amplify activity and selectivity by engaging distal CRBN or target residues. This modular interplay allows systematic tuning of degrader potency and specificity.

Western blots for Supplementary Figures

Supplementary Figure 9:

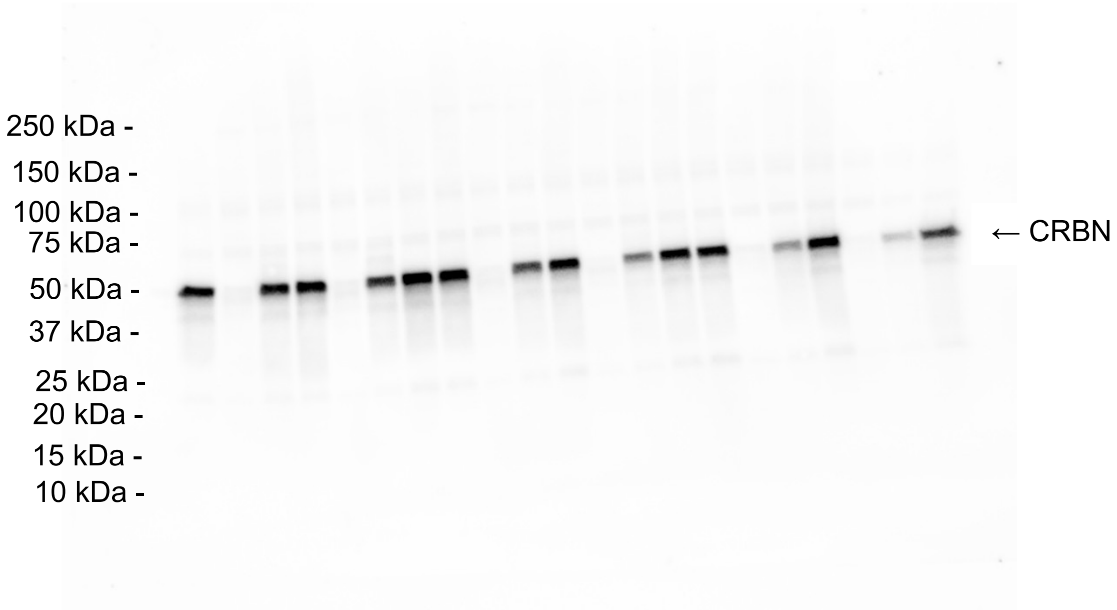

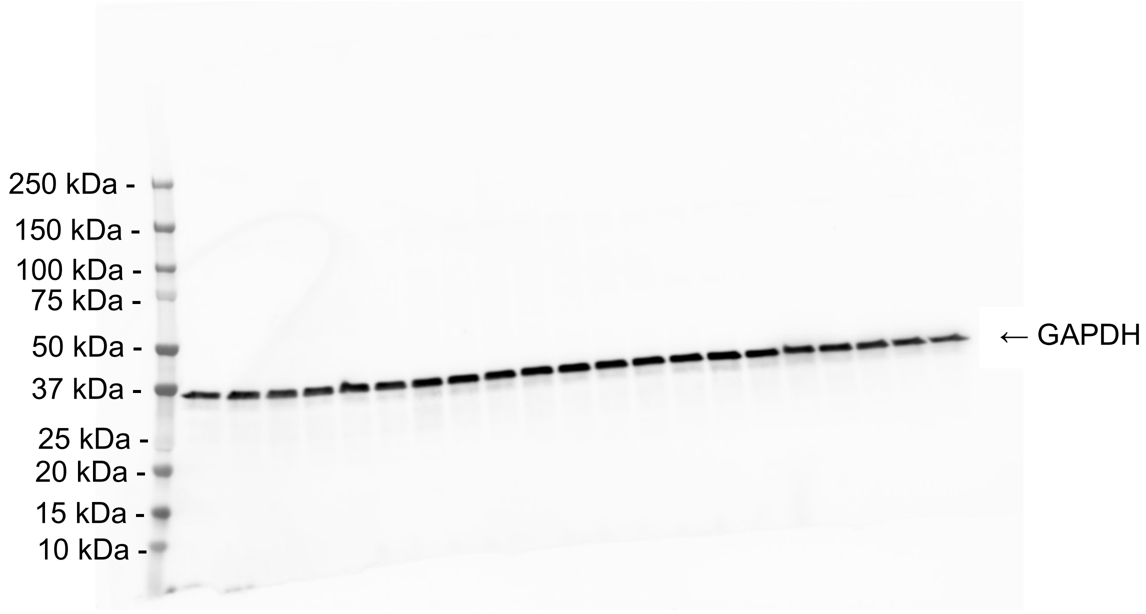

Supplement: Supplementary file 1 — Supplementary Information [file 41467_2025_65094_MOESM1_ESM.pdf]
