# Supplementary material for: A degron-mimicking molecular glue drives CRBN homo-dimerization and degradation
Source: Nat Commun. 2025 Nov 19;16:10157. doi: 10.1038/s41467-025-65094-3 (PMC12630686; doi:10.1038/s41467-025-65094-3)
Supplement: Supplementary file 2 — Description of Additional Supplementary Files [file 41467_2025_65094_MOESM2_ESM.docx]

**Description of Additional Supplementary Files**

**Supplementary Movie 1: Dynamight flexibility analysis.**

The movie represents a circular journey through the latent space, following the white-to-red gradient indicated in Supplementary Figure 4.
